# Statin use and ocular inflammatory disease risk

**DOI:** 10.1186/1869-5760-3-8

**Published:** 2013-01-11

**Authors:** Jacob J Yunker, Gerald McGwin, Russell W Read

**Affiliations:** 1 Department of Ophthalmology, School of Medicine, University of Alabama at Birmingham, 700 18th Street South, Suite 601, Birmingham, AL, 35233, USA; 2 Retina Consultants of Alabama, 700 18th Street South, Birmingham, AL, 35233, USA; 3 Department of Epidemiology, School of Public Health, University of Alabama at Birmingham, 700 18th Street South, Birmingham, AL, 35233, USA; 4 Department of Surgery, School of Medicine, University of Alabama at Birmingham, 700 18th Street South, Birmingham, AL, 35233, USA; 5 Department of Pathology, School of Medicine, University of Alabama at Birmingham, 700 18th Street South, Birmingham, AL, 35233, USA

**Keywords:** Uveitis, Statins, Epidemiology, Veteran

## Abstract

**Background:**

This study aims to evaluate the effect of oral statin medication use on the subsequent development of ocular inflammatory disease (OID). A retrospective nested case–control study was carried out on patient records from the Birmingham Veterans Affairs Medical Center. All male patients with a new diagnosis of OID over a 5-year period were included. Ten control subjects (without OID) were age-matched to each OID case. Prescription files of all subjects were queried for statin use. Information on selected comorbid medical conditions was also obtained. Conditional logistic regression was used to calculate odds ratios (ORs) and 95% confidence intervals (CIs) for risk of OID development in the context of statin use, controlling for comorbid conditions.

**Results:**

Ninety-two incident cases of OID were identified. A trend toward a reduction in the risk of new OID development was found in patients that used statins compared to those that did not (OR 0.50, 95% CI 0.20 to 1.23, *p* = 0.13). The longer the duration of statin use, the greater is the effect.

**Conclusions:**

Use of oral statins may be associated with a reduced risk for the development of OID. This reduced risk increases with increasing duration of use. Larger clinical studies would be required to definitively establish the effectiveness of statins in lowering the incidence of OID.

## Background

Uveitis refers to inflammation of the uveal tract. Uveitis is a frequent cause of ocular complications with subsequent visual impairment [1]. Two studies of patient populations in the west coast of the USA have estimated that the incidence of uveitis ranges from 25.6 to 52 cases per 100,000 person-years and the prevalence ranges from 69 to 115 cases per 100,00 persons [2,3]. Interestingly, the lower estimate of incidence and prevalence above are obtained from a study of Veterans Affairs (VA) patients in the Pacific Northwest, pertinent to the current study's patient population. As uveitis accounts for up to 10% of blindness in the USA and up to 15% worldwide [4], it is a disproportionate cause of visual disability in light of its frequency. Scleritis is inflammation of the sclera, and similar to uveitis, it is primarily due to autoimmune disease [5-8]. Collectively, uveitis and scleritis may be referred to as ocular inflammatory diseases (OID). Treatment for autoimmune OID consists of suppression of inflammation with corticosteroids or immunomodulatory/steroid-sparing medications. Antimetabolite medications are the most commonly used after corticosteroids [9] and are effective in up to approximately two-thirds of patients [10-12]. Alkylating agents are more effective, but may carry with them greater risks as well [9,13]. Newer agents such as biologic response modifiers hold great promise for the treatment of OID, but may also have increased risks [9].

The use of 3-hydroxy-3-methylglutaryl coenzyme A reductase inhibitors (the so-called ‘statins’) has been shown to be beneficial in reducing cardiovascular morbidity and mortality [14]. In addition to reducing serum cholesterol levels, statins are also believed to modify atherosclerosis via anti-inflammatory pathways [15]. Multiple studies have suggested that these modulatory effects of statins may be therapeutic in systemic autoimmune disorders [16]. Various studies have examined the possible impact of statins in ophthalmic conditions, including age-related macular degeneration [17-23], glaucoma [24], and diabetic retinopathy [25-27]. Although the role of statins in ocular disease is not fully understood, given the numerous mechanisms by which statins may modify the inflammatory response, we sought to investigate the potential impact of statin use on OID.

## Results

Ninety-two incident cases of OID were identified. Demographic and medical characteristics of all subjects are detailed in Table 1. By design, the mean age of both cases and control subjects was equivalent. There were similar numbers of each race among the two groups; however, the number for whom race was not known was twice as high among the control group. Those with OID were more likely also to have diabetes and hypertension, whereas the prevalence of lipid metabolism disorders was not statistically different between groups.

**Table 1:** Characteristics of OID cases and control subjects

	**OID cases**	**Control subjects**	***p *value**
Number of subjects	92	920	
Mean age (year)s	58.2	58.1	0.97^a^
Ethnicity			0.0018^b^
White	48 (52.2)	378 (41.1)	
African-American	30 (32.6)	207 (22.5)	
Other	0 (0)	6 (0.6)	
Unknown	14 (15.2)	329 (35.8)	
Medical characteristics			
Diabetes	24 (26.1)	95 (10.3)	<0.0001^b^
Lipid metabolism disorders	7 (7.6)	53 (5.8)	0.47^b^
Hypertension	32 (34.8)	185 (20.1)	0.0011^b^
Cardiovascular disease	12 (13.0)	141 (15.3)	0.56^b^
Cerebrovascular disease	2 (2.2)	47 (5.1)	0.21^b^
Arterial disease	3 (3.3)	32 (3.5)	0.91^b^

Values are expressed as number (percentage) of individuals unless otherwise indicated. OID, ocular inflammatory disease. ^a^Paired *t* test; ^b^McNemar's test.

Table 2 details the statin use characteristics among OID cases and control subjects as well as the unadjusted and adjusted ORs. Stratified analyses for the effect of comorbidities (ischemic heart disease, cerebrovascular disease, lipid metabolism disorders, hypertension, diseases of the arteries, arterioles, and capillaries, and diabetes) on the association between statin use and OID revealed no evidence of such effect (data not shown). Following adjustment for diabetes, lipid metabolism disorders, hypertension, cardiovascular disease, cerebrovascular disease, and arterial disease, a twofold reduction in the risk of OID development was associated with any history of statin use (OR, 0.50; 95% CI, 0.20 to 1.23), although this did not reach statistical significance (*p* = 0.13). The reduction in the risk of OID was greater as the duration of statin use increased, as seen in Table 2.

**Table 2:** Statin medication use characteristics among uveitis cases and control subjects

	**Uveitis cases**	**Control subjects**	**OR (95% CI)**	**OR (95% CI)**^**a**^	***p *value**
Number of subjects	92	920			
Statin use					
No	84 (91.3)	819 (89.0)	1.00 (reference)	1	
Yes	8 (8.7)	101 (11.0)	0.79 (0.38 to 1.63)	0.50 (0.20 to 1.23)	0.13
Current use	4 (4.4)	53 (5.8)	0.76 (0.28 to 2.06)	0.46 (0.15 to 1.46)	0.19
Past use	4 (4.4)	48 (5.2)	0.83 (0.30 to 2.26)	0.54 (0.18 to 1.67)	0.29
Duration of use (month)					
No use	84 (91.3)	819 (89.0)	1.00 (reference)	1	
<12	5 (5.4)	44 (4.8)	1.10 (0.45 to 2.70)	0.74 (0.27 to 2.07)	0.57
12 to 23	2 (2.2)	23 (2.5)	0.86 (0.21 to 3.50)	0.45 (0.10 to 2.01)	0.29
>23	1 (1.1)	34 (3.7)	0.31 (0.04 to 2.21)	0.19 (0.02 to 1.52)	0.12

Values are expressed as number (percentage) of individuals unless otherwise indicated. Analyses are for OID versus no OID. OID, ocular inflammatory disease; OR, odds ratio. ^a^Adjusted for diabetes, lipid metabolism disorders, hypertension, cardiovascular disease, cerebrovascular disease, and arterial disease.

## Discussion

There are several mechanisms by which statins might exhibit anti-inflammatory effects in the eye [28]. Statins are known to inhibit the activation of Rho guanosine triphosphatase (GTPase), a key molecule in the endothelial ICAM-1-mediated pathway that facilitates lymphocyte migration [29-31]. Statins thus inhibit interactions between leukocytes and endothelial cells, preventing leukocyte transmigration from the vasculature, across the blood-retinal barrier [29,32-34]. Endothelial cell nitric oxide synthase expression is up-regulated in the presence of statins, leading to higher levels of nitric oxide, which has protective effects on endothelial cells [35]. Statins also inhibit the formation of oxygen free radicals by endothelial cells [36,37]. Thus, statins may act to stabilize the blood-ocular and blood-retinal barrier, transgression across which enables inflammatory mediators and immune activator cells to enter the anterior chamber, vitreous cavity, and retinal tissues. In addition, statins reduce a number of key inflammatory cytokines, including IL-6, IL-8, and TNF [38,39]. Finally, statins decrease T cell activation by inhibition of GTPase activity [40-43].

The effect of statins in animal models of uveitis has been examined. In a mouse model using B10.RIII mice, a strain that is highly susceptible to experimental autoimmune uveitis (EAU) and typically manifests severe disease, Thomas and colleagues [44] found that atorvastatin administered orally did not have a significant effect on measured levels of inflammatory cytokines, although they did observe a mildly reduced level of histological inflammation in the group receiving high-dose (10 mg/kg/day) treatment. Similarly, Gegg and colleagues [45] reported that neither atorvastatin nor lovastatin administered orally demonstrated a significant therapeutic effect. However, parenteral lovastatin (20 mg/kg/day) suppressed clinical ocular pathology, retinal vascular leakage, and leukocyte infiltration into the retina. In a rat model of uveitis induced with S-antigen, Kohno and coworkers [46] demonstrated a therapeutic effect for both atorvastatin (10 mg/kg/day) administered orally and lovastatin (2 mg/kg/day) administered intraperitoneally. These studies suggest that the effect of statins varies by route of administration, dosage, as well as across different models of EAU. This data indicates that the potential beneficial effects of statins in EAU may require doses greater than those used in the routine treatment of hyperlipidemia in humans. For example, the highest recommended dose of atorvastatin in humans is 80 mg/day, which for a 70-kg male adult is 1.14 mg/kg/day, ninefold lower than the effective dose used by Kohno and colleagues [46]. In the current study, it is likely that the majority of filled statin prescriptions were for simvastatin, as this was the first line medication on the BVAMC formulary for hyperlipidemia during the study period. The BVAMC data set did not include medication dosage information, so we are unable to comment on an association between dosage and OID protection.

The current study has numerous limitations. First, the database consisted exclusively of an older veteran population, and the study was limited entirely to male subjects. This may have introduced a population selection bias in that the incidence of OID in this somewhat homogenous group may be higher or lower than that found among other populations [2,3], including women. However, the homogeneous study population makes these results all the more applicable to the demographic of Caucasian males. Second, the diagnoses of OID were made by individual physicians without the use of standardized criteria, which could introduce differences relative to other studies. However, there is no reason to expect the diagnosis of OID to have been biased by the use of statins. Multiple forms of OID (Table 3) were combined for analysis, with varying etiologies and effects on ocular health. This potpourri of OID introduces additional variability in comparison with other study populations. In addition, if a patient presented as a new VA patient but with longstanding OID, that patient would have been classified as an incident case as opposed to a prevalent case. Because of the nature of the database, adjudication of each OID case to verify whether it was incident or prevalent was not possible. Third, the diagnoses were subject to miscoding into ICD-9CM codes; again, there is no reason to suspect that this would introduce bias. Fourth, statin use was defined on the basis of a filled prescription within the BVAMC pharmacy service. In this scenario, a patient with a statin prescription record but no matching fill record would be classified as a non-statin user, even if he did use statins by filling the prescription outside the BVAMC system. Such misclassification, however, would only bias toward the null. Additionally, since more than 90% of statin prescriptions were filled at the BVAMC, this is unlikely to have produced a significant effect. We also did not have information on statin use outside the Veterans Affairs system and therefore could have underestimated some subjects' duration of use. As long as such misclassification is not differential according to case status, then the effect is likely to be minimal. Fifth, race was unknown for a large portion of the control subjects. Additional potentially confounding demographic factors, such as smoking, which has recently been shown to be associated with uveitis [47,48], were also not available. 

**Table 3:** ICD-9CM codes used

**ICD-9**	**Diagnosis**
136.10	Behçet's disease
360.02	Panophthalmitis
360.11	Sympathetic uveitis
360.12	Panuveitis
360.19	Phacoanaphylactic uveitis
362.18	Retinal vasculitis
363.00	Focal chorioretinitis, unspecified
363.01	Focal choroiditis and chorioretinitis, justapapillary
363.03	Focal choroiditis and chorioretinitis of other posterior pole
363.04	Focal choroiditis and chorioretinitis, peripheral
363.05	Focal retinitis and retinochoroiditis, justapapillary
363.06	Focal retinitis and retinochoroiditis, macular or paramacular
363.07	Focal retinitis and retinochoroiditis of other posterior pole
363.08	Focal retinitis and retinochoroiditis, peripheral
363.10	Disseminated chorioretinitis, unspecified
363.11	Disseminated choroiditis and chorioretinitis, posterior pole
363.12	Disseminated choroiditis and chorioretinitis, peripheral
363.13	Disseminated choroiditis and chorioretinitis, generalized
363.14	Disseminated retinitis and retinochoroiditis, metastatic
363.15	Disseminated retinitis and retinochoroiditis, pigment epitheliopathy
363.20	Chorioretinitis, unspecified
363.21	Pars planitis
363.22	Harada's disease
364.00	Acute and subacute iridocyclitis
364.01	Primary iridocyclitis
364.02	Recurrent iridocyclitis
364.03	Secondary iridocyclitis, infectious
364.04	Secondary iridocyclitis, noninfectious
364.10	Chronic iridocyclitis
364.11	Chronic iridocyclitis in diseases classified elsewhere
364.20	Certain types of iridocyclitis
364.21	Fuchs' heterochromic iridocyclitis
364.22	Glaucomatocyclitic crises
364.23	Lens-induced iridocyclitis
364.24	Vogt-Koyanagi syndrome
364.30	Unspecified iridocyclitis
365.62	Glaucoma associated with ocular inflammations
366.32	Cataract in inflammatory disorders
379.00	Scleritis, unspecified
379.01	Episcleritis periodica fugax
379.02	Nodular episcleritis
379.03	Anterior scleritis
379.04	Scleromalacia perforans
379.05	Scleritis with corneal involvement
379.06	Brawny scleritis
379.07	Posterior scleritis
379.09	Other scleritis

Strengths of this study include its nested case–control design, which allowed for the identification of statin use before the diagnosis of OID. Additionally, an electronic prescription record was utilized, obviating the need to rely on self-reported medication use which may be historically inaccurate.

## Conclusions

In summary, the data presented demonstrate a possible reduction in the risk of the development of OID in patients using statins, a reduction that becomes more pronounced with increasing duration of use. This effect did not reach statistical significance, with a *p* value of 0.13 for any use of statins. It is important not to overemphasize this finding given the non-signifcance, but it is important not to reject an effect out of hand in an underpowered study. A twofold reduction in risk, with a *p* value trending toward significance, may be statistically insignificant, but still clinically relevant. To fully address the question of the effect of statin use on OID risk, evaluation of larger databases with longer follow-up periods, such as insurance company claim files where both disease and pharmacy data are maintained, could provide a definitive answer. A randomized clinical trial would be necessary to establish whether statins would be a useful addition to the therapeutic arsenal against OID.

## Methods

### Study population and data source

The Birmingham Veterans Affairs Medical Center (BVAMC) is a 134-bed acute tertiary care medical facility and referral center in Alabama. All patients who had at least one visit (inpatient or outpatient) at the BVAMC between 01 January 1997 and 31 December 2001 were eligible for study inclusion. Females were excluded as they represented such a small proportion of the patient population (10.8%) that meaningful analyses were impossible.

The BVAMC provided data files containing demographic information (age, sex, race) and clinical and medication information for each patient. The clinical file contained a description of each diagnosis made at the BVAMC during inpatient and outpatient visits and the diagnosis date. All diagnoses were coded using the International Classification of Diseases, Ninth Revision, Clinical Modification (ICD-9CM). The medication file contained information on each medication prescribed during each patient visit. This file also contained the prescription date and the date the prescription was filled. For both the clinical and medication files, the information provided pertained to all diagnoses and medications over the course of each patient's history with the BVAMC and not just those that occurred in 1997 to 2001. All data received from the BVAMC contained no information that would allow patients to be identified. The Birmingham Veterans Affairs Medical Center IRB approved the study prior to data collection and analysis. All methods complied with HIPAA guidelines. These data files were locked for the indicated time period, and no additional data on the BVAMC population has been made available since 2001, limiting the study period to that indicated above.

### Study design

Within the study population, a nested case–control study was conducted. Cases of OID were defined using the ICD-9CM codes indicated in Table 3. Information on the OID diagnosis date was procured and will heretofore be referred to as the index date. Because this study addressed the association between statin use and the incidence of OID, patients who had an OID diagnosis before the observation period (1997 to 2001) of the study (prevalent cases) were excluded.

Controls were randomly selected from the study population who did not have an OID diagnosis by the end of the observation period. To be considered an eligible control for a given case, the control must have had an encounter with the BVAMC (inpatient or outpatient) on or before the index date of the matched case. Ten controls were selected for each case and matched on age (±1 year). Each control was assigned the index date associated with their matched case.

The prescription file was queried for the presence of filled statin (atorvastatin, cerivastatin, fluvastatin, pravastatin, simvastatin, lovastatin) prescriptions. Only those prescriptions that were filled before the index date for each matched set of cases and controls were considered. Patients were classified as being either statin users or non-statin users. Statin users were further classified as current or past users, with the former being those who had a statin prescription filled within 6 months before the index date and the latter being those whose last prescription fill date was more than 6 months before the index date.

Information on the presence of the following conditions was extracted from the clinical data file: ischemic heart disease (ICD-9CM codes 410 though 414), cerebrovascular disease (ICD-9CM codes 430 though 438), lipid metabolism disorders (ICD-9CM code 272), hypertension (ICD-9CM codes 401 though 405), diseases of the arteries, arterioles, and capillaries (ICD-9CM codes 440 though 448), and diabetes (ICD-9CM code 250). For the purposes of analysis, only those diagnoses that were recorded before the index date were considered.

### Statistical analysis

Conditional logistic regression was used to calculate an odds ratio (OR) and 95% confidence interval (CI) for the association between any statin use and the risk of developing OID. Odds ratios and 95% confidence intervals were also estimated for current and past statin users relative to non-users and according to time since first prescription. Stratified analyses were conducted to determine if ischemic heart disease, cerebrovascular disease, lipid metabolism disorders, hypertension, diseases of the arteries, arterioles, and capillaries, and diabetes modified the association between statin use and OID. For both unstratified and stratified analyses, estimates were obtained without and with adjustment for diabetes, lipid metabolism disorders, hypertension, ischemic heart disease, cerebrovascular disease, and arterial disease.

## Competing interests

The authors declare that they have no competing interests.

## Authors’ contributions

JJY participated in the conceptualization and design of the study, carried out the ICD code review and selection, and drafted the initial manuscript. GM participated in the conceptualization and design of the study and carried out all statistical analyses. RWR participated in the conceptualization and design of the study and wrote the final draft of the manuscript. All authors read, participated in revising, and approved the final manuscript.

## Authors’ information

RWR is a Research to Prevent Blindness physician scientist.
